# CGRP Signaling via CALCRL Increases Chemotherapy Resistance and Stem Cell Properties in Acute Myeloid Leukemia

**DOI:** 10.3390/ijms20235826

**Published:** 2019-11-20

**Authors:** Tobias Gluexam, Alexander M. Grandits, Angela Schlerka, Chi Huu Nguyen, Julia Etzler, Thomas Finkes, Michael Fuchs, Christoph Scheid, Gerwin Heller, Hubert Hackl, Nathalie Harrer, Heinz Sill, Elisabeth Koller, Dagmar Stoiber, Wolfgang Sommergruber, Rotraud Wieser

**Affiliations:** 1 Division of Oncology, Department of Medicine I, Medical University of Vienna, Waehringer Guertel 18-20, 1090 Vienna, Austria; tobias.gluexam@meduniwien.ac.at (T.G.); alexander.grandits@meduniwien.ac.at (A.M.G.); angela.schlerka@meduniwien.ac.at (A.S.); chi.nguyen@meduniwien.ac.at (C.H.N.); julia.etzler@gmx.net (J.E.); thomas.finkes@gmx.net (T.F.); gerwin.heller@meduniwien.ac.at (G.H.); 2 Comprehensive Cancer Center, Spitalgasse 23, 1090 Vienna, Austria; 3 Department I of Internal Medicine, Center for Integrated Oncology Aachen Bonn Cologne Duesseldorf, University of Cologne, Kerpener Str. 62, 50937 Cologne, Germany; michael.fuchs@uk-koeln.de (M.F.); christoph.scheid@uk-koeln.de (C.S.); 4 Institute of Bioinformatics, Biocenter, Medical University of Innsbruck, Innrain 80, 6020 Innsbruck, Austria; hubert.hackl@i-med.ac.at; 5 Department for Cancer Research, Boehringer Ingelheim RCV GmbH & Co KG, Dr. Boehringer-Gasse 5-11, A-1121 Vienna, Austria; nathalie.harrer@boehringer-ingelheim.com; 6 Division of Hematology, Medical University of Graz, Auenbruggerplatz 38, 8036 Graz, Austria; heinz.sill@medunigraz.at; 7 Third Medical Department, Hanusch Hospital, Heinrich Collinstrasse 30, 1140 Vienna, Austria; elisabeth.koller@wgkk.at; 8 Institute of Pharmacology, Center for Physiology and Pharmacology, Medical University of Vienna, Waehringer Strasse 13A, 1090 Vienna, Austria; dagmar.stoiber@kl.ac.at; 9 Division Pharmacology, Department Pharmacology, Physiology and Microbiology, Karl Landsteiner University of Health Sciences, Dr.-Karl-Dorrek-Straße 30, 3500 Krems, Austria; 10 Department of Biotechnology, University of Applied Sciences, Helmut-Qualtinger-Gasse 2, 1030 Vienna, Austria; w.sogru@outlook.com

**Keywords:** CALCRL, CGRP, AML, relapse, chemotherapy resistance, leukemic stem cells, olcegepant

## Abstract

The neuropeptide CGRP, acting through the G-protein coupled receptor CALCRL and its coreceptor RAMP1, plays a key role in migraines, which has led to the clinical development of several inhibitory compounds. Recently, high *CALCRL* expression has been shown to be associated with a poor prognosis in acute myeloid leukemia (AML). We investigate, therefore, the functional role of the CGRP-CALCRL axis in AML. To this end, *in silico* analyses, human AML cell lines, primary patient samples, and a C57BL/6-based mouse model of AML are used. We find that *CALCRL* is up-regulated at relapse of AML, in leukemic stem cells (LSCs) versus bulk leukemic cells, and in LSCs versus normal hematopoietic stem cells. CGRP protects receptor-positive AML cell lines and primary AML samples from apoptosis induced by cytostatic drugs used in AML therapy, and this effect is inhibited by specific antagonists. Furthermore, the CGRP antagonist olcegepant increases differentiation and reduces the leukemic burden as well as key stem cell properties in a mouse model of AML. These data provide a basis for further investigations into a possible role of CGRP-CALCRL inhibition in the therapy of AML.

## 1. Introduction

*Calcitonin Receptor Like Receptor* (*CALCRL*) codes for a G-protein-coupled seven-transmembrane domain receptor with important roles in normal physiology and disease [1]. Co-expression of one of three single transmembrane domain coreceptors, namely, receptor activity modifying protein (RAMP) 1, RAMP2, or RAMP3, is required both for its expression at the cell surface and binding of its peptide ligands [2]. The CALCRL/RAMP1 complex binds the 37 amino acid neuropeptide, calcitonin gene-related peptide (CGRP), while the CALCRL/RAMP2 and CALCRL/RAMP3 complexes bind adrenomedullin (ADM) [1,2,3]. Ligand binding effects a rise in cyclic AMP (cAMP) levels and activation of protein kinase A [1,3,4]. CGRP is a multifunctional peptide involved, for example, in the regulation of blood pressure, wound healing, and neurotransmission [1,3,5] and, additionally, contributes to various diseases [1]. Most prominently, CGRP plays a causative role in migraines, which has led to the development of small molecule antagonists and function-blocking monoclonal antibodies, some of which have been approved, or are under review, for clinical use [5].

CGRP and its receptor also have been implicated in the pathogenesis of malignant diseases. The expression of CGRP, the closely related peptide CGRP2, and *CALCRL* was increased in some tumor types compared to the corresponding healthy tissues [3,6,7]. CGRP stimulated proliferation and inhibited apoptosis of both normal and malignant cells [3,6,8,9,10,11], and promoted migration and invasiveness of some carcinoma cell lines [3]. Furthermore, CGRP may foster tumor growth through its ability to promote angiogenesis [11]. Accordingly, knockdown of *CALCB* (which encodes CGRP2 and is activated by the Ewing sarcoma associated fusion protein EWSR1-FLI) or *RAMP1* decreased growth of Ewing sarcoma cell lines *in vitro* and in a mouse xenograft model, and the small molecule CGRP antagonists MK-3207 and olcegepant reduced colony and sphere formation by Ewing sarcoma cells [6].

Acute myeloid leukemia (AML) is an aggressive hematopoietic malignancy with an annual incidence of 3–8/100.000 and a median age of onset of around 67 years [12,13]. It is organized in a hierarchical manner, with the bulk of the leukemic cell mass being derived from mostly quiescent leukemic stem cells (LSCs) [14,15]. AML results from genetic and epigenetic alterations, which provide the malignant cells with growth and survival advantages by causing quantitatively and/or qualitatively aberrant gene expression [16,17,18,19,20,21,22]. Recurrent molecular alterations are useful prognostic markers [19,20,21,22] and, in addition, represent targets for rationally designed therapies, some of which recently have been approved for clinical use [23,24]. Nevertheless, chemotherapy based on cytosine arabinoside (araC) and an anthracycline like daunorubicin remains the mainstay of AML treatment. It leads to complete remissions in 50–80% of patients, yet the majority eventually relapse with, and die from, largely chemotherapy-resistant disease [25,26,27]. Malignant cells re-growing at the time of relapse are mono- or oligo-clonal [28]. They may have survived the initial chemotherapy by hiding away in a protective niche [29,30] and/or due to additional resistance-conferring molecular alterations [27,28,31,32], some of which may, in fact, act by allowing leukemic cells to better interact with stroma. Molecular changes acquired, or selected for, between diagnosis and relapse of AML can be expected, therefore, to contribute to therapy resistance in a prominent manner. However, even though a large proportion of patients exhibited genetic alterations of various types that were newly acquired at relapse, few if any of these alterations were associated with relapse in a recurrent manner [27]. To contrast, changes in promoter methylation and the transcription of specific genes did occur in substantial proportions of patients [33,34]. Specifically, in our own study on paired diagnosis-relapse samples from 11 patients with cytogenetically normal AML, 536 and 551 unique genes were found to be significantly up- and down-regulated at relapse, respectively [34]. This relapse-associated gene expression signature was significantly enriched for gene expression profiles defining AML LSCs and/or linked to poor outcome in AML, confirming the notion that genes deregulated at relapse are related to stemness and chemotherapy resistance [34]. One of the top up-regulated genes in the relapse signature was *CALCRL* [34], a finding recently confirmed by others [35]. Nerve fibers immunoreactive for CGRP are present, and often associated with blood vessels, in the bone marrow (BM) [36,37], suggesting that both normal and malignant hematopoietic cells are exposed to this CALCRL ligand. Functional CALCRL and RAMP1 were expressed in immature hematopoietic cells, but not in mature myeloid cells [38,39], and CGRP stimulated proliferation and inhibited apoptosis of hematopoietic cells [9,40]. Targeted deletion of *Ramp1*, the only *Ramp* gene expressed in the hematopoietic stem cell (HSC) enriched murine Lin^−^ Sca-1^+^ Kit^+^ (LSK) population, did not affect steady state hematopoiesis in mice, but reduced various hematopoietic cell populations under stress conditions [39]. Recently, *CALCRL* was identified as part of a 3-gene signature associated with poor outcomes in AML [35], and the prognostic relevance of CALCRL in AML was corroborated both on the mRNA and the protein level [41]. However, little if any experimental data demonstrating a specific function of CALCRL in AML cells were presented in these reports. Here, we use publicly available data sets to confirm the association between high *CALCRL* mRNA levels and poor outcomes of AML, and to show elevated *CALCRL* expression in leukemic versus normal hematopoietic stem cells. Importantly, we also provide experimental evidence that CGRP increases chemotherapy resistance in AML cells, and its antagonist olcegepant, applied as a single agent, decreases the leukemic burden and AML stem cell properties *in vivo*.

## 2. Results

### 2.1. Expression of CALCRL in Primary Samples from Patients with AML is Associated with Increased Resistance to Chemotherapy

**Figure 1:**
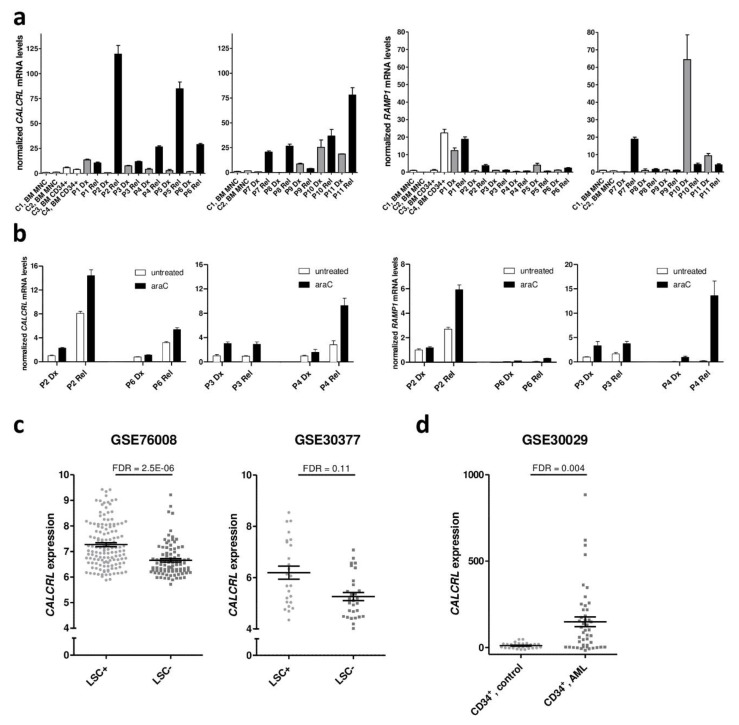
*CALCRL* expression is elevated at relapse of AML and in leukemic stem cells, and induced by araC. (**a**) Expression of *CALCRL* and *RAMP1* in paired samples from diagnosis (Dx) and relapse (Rel) of AML was determined by qRT-PCR. Normalization to β-2-microglobulin and calibration to sample C1 was performed using the ΔΔC_T_ method. C1–C4, healthy control samples 1–4; BM, bone marrow; MNC, mononuclear cells; P1–P11, patients 1–11. Means + SD from technical replicates. (**b**) Expression of *CALCRL* and *RAMP1* in paired samples from diagnosis (Dx) and relapse (Rel), cultured in the presence or absence of araC for 2 days, was measured as in (**a**). (**c**) Expression of *CALCRL* in stem cell enriched (LSC+) *versus* stem cell depleted (LSC–) leukemic cell populations contained in data sets GSE76008 and GSE30377. (**d**) Expression of *CALCRL* in CD34^+^ cell populations from AML *versus* healthy donors, contained in data set GSE30029. (**c**,**d**) Data were used as provided in the respective data sets (log2 transformed or not). False discovery rate (FDR) was determined using the lmFit function of the R package Limma.

To confirm our previous finding that *CALCRL* is up-regulated at relapse of AML [34], qRT-PCR was performed on 11 paired samples from the times of diagnosis and relapse. BM mononuclear cells (MNCs) and BM CD34^+^ cells from two healthy donors each were included for comparison. Among healthy donors, *CALCRL* mRNA expression was higher in the hematopoietic stem and progenitor cell enriched CD34^+^ fraction than in bulk MNCs (*p* = 0.058 (just short of significance probably due to small sample numbers); Figure 1a). At diagnosis of AML, *CALCRL* expression was elevated compared to normal BM MNCs (*p* = 0.0293). At relapse, *CALCRL* mRNA levels were further increased compared to the matched diagnostic samples (*p* = 0.0176); consequently, they were also higher than in normal BM MNCs (*p* = 0.0053; Figure 1a). Regarding the genes encoding the coreceptors RAMP1, 2, and 3, *RAMP1* was expressed in all AML samples, but with no clear pattern between the two time points (*p* = 0.564; Figure 1a). To contrast, *RAMP3* was expressed only in 3/12 analyzed AML samples (one from diagnosis, two from relapse; Figure S1a), and *RAMP2* could not be detected at all. Thus, the predominant CALCRL-containing receptor in AML is the CGRP receptor, composed of CALCRL and RAMP1; adrenomedullin receptors containing CALCRL and RAMP3 may be present in a minor proportion of patients.

Because *CALCRL* was up-regulated at relapse, i.e., in cells re-accumulating after chemotherapy, we next asked whether exposure to araC, the mainstay of AML treatment, would affect *CALCRL* expression. Diagnosis and relapse samples from patients P2, P3, P4, and P6 were cultured in the presence or absence of araC for 48 h and subjected to qRT-PCR. Up-regulation of *CALCRL* at relapse was maintained in the cells after short term culture. Furthermore, araC substantially and significantly increased *CALCRL* expression, both in diagnostic and relapse samples (*p* = 0.0019), and a similar, albeit non-significant pattern was observed for *RAMP1* (*p* = 0.219; Figure 1b).

To collect further information about the expression and potential prognostic relevance of *CALCRL* and *RAMP1* in normal hematopoiesis and in AML, several publicly available data sets were used. Agreeing with the above described findings (Figure 1a), in healthy donors, *CALCRL* mRNA levels were significantly higher in HSCs and progenitors than in lineage marker positive cells (GSE30377 [42]; Figure S1b). Regarding AML, *CALCRL* mRNA levels were elevated in LSC enriched versus LSC depleted cell populations (GSE76008 [22]; GSE30377 [42]; Figure 1c). *CALCRL* expression also was stronger in CD34^+^ (stem cell enriched) cell populations from AML versus those from healthy donors (GSE30029 [43]; Figure 1d). Moreover, high *CALCRL* expression was a prognostic parameter for poor overall survival in 7/7 AML patient cohorts contained in data sets GSE12417 [18], GSE6891 [44], GSE37642 [45], GSE71014 [46], and TCGA [19] (Table 1, Table S1, Figure S1c). In the majority of these, this association was confirmed in multivariable models (Table 1). In contrast, *RAMP1* was not differentially expressed between the above mentioned groups, and its association with survival was inconsistent and, with one exception, lost in multivariable analysis (Table S1).

To summarize, high *CALCRL* expression was associated with stemness, relapse, and poor outcome of AML, and higher expression of *CALCRL* in LSCs versus HSCs suggests the existence of a potential therapeutic window for CALCRL inhibitors. Even though similar associations were not observed for *RAMP1*, it was consistently expressed in AML cells. Thus, signaling through the CGRP-CALCRL axis may contribute to chemotherapy resistance in AML.

### 2.2. CGRP Increases the Resistance of Human AML Cell Lines to Drugs Used in the Treatment of AML

**Figure 2:**
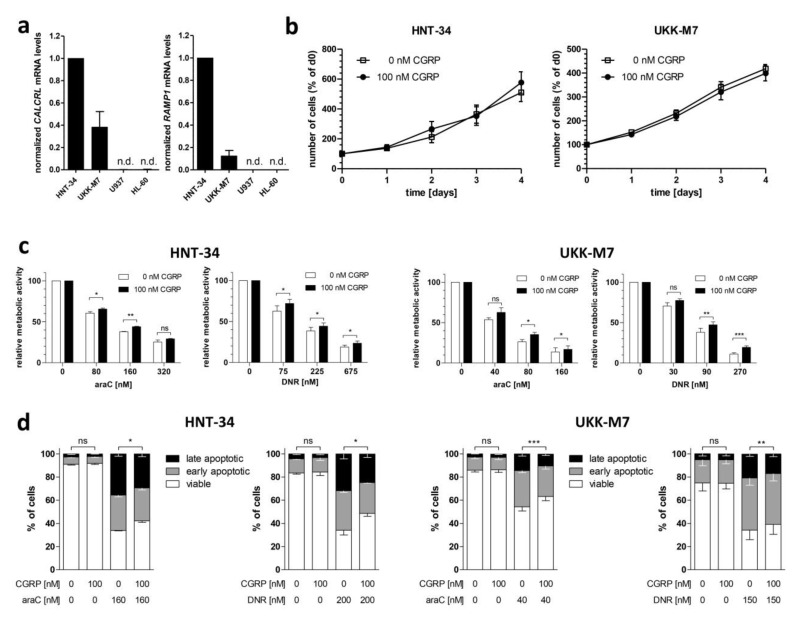
CGRP increases the resistance of receptor positive human AML cell lines to drugs used in the treatment of AML. (**a**) Expression of *CALCRL* and *RAMP1* in the human myeloid cell lines HNT-34, UKK-M7, U937, and HL-60 was determined by qRT-PCR. Normalization to β-2-microglobulin mRNA levels and calibration to HNT-34 was performed using the ΔΔC_T_ method. n.d., not detectable. Means + SEM from 3 biological replicates. (**b**) Proliferation of HNT-34 and UKK-M7 cells in the absence or presence of 100 nM CGRP. Means +/− SEM from 3 biological replicates. (**c**,**d**) CGRP protects HNT-34 and UKK-M7 cells from araC- and daunorubicin (DNR)-induced apoptosis. Cells were pre-incubated with or without 100 nM CGRP for 1 h prior to addition of the indicated concentrations of cytostatic drugs. Means +/− SEM from 3–5 biological replicates. ns, not significant, * *p* < 0.05, ** *p* < 0.01, *** *p* < 0.001, paired Student’s two-tailed t-test. (**c**) CellTiter-Glo metabolic activity assay. (**d**) AnnexinV/DAPI assay.

To investigate the effects of CGRP on human AML cells, the cell lines HNT-34 [47] and UKK-M7, newly established from a patient with AML-M7 (see Methods for further details), were used. Both cell lines robustly express *CALCRL* and *RAMP1,* as determined by qRT-PCR (Figure 2a). Incubation with 100 nM CGRP for up to 4 days did not affect their proliferation in a suspension culture (Figure 2b). However, pre-treatment with CGRP significantly increased their metabolic activity (a proxy for viability) after exposure to various doses of araC or daunorubicin (Figure 2c). This indicated that CGRP-CALCRL signaling indeed increased resistance to the drugs used to treat AML, as hypothesized based on the expression data described above. These results were corroborated through the AnnexinV/DAPI assay: CGRP pre-treatment significantly increased the number of viable HNT-34 and UKK-M7 cells in the presence of araC or daunorubicin (Figure 2d, Figure S2).

### 2.3. Genetic Inhibition of CALCRL Counteracts the CGRP-Induced Increase in Chemotherapy Resistance

**Figure 3:**
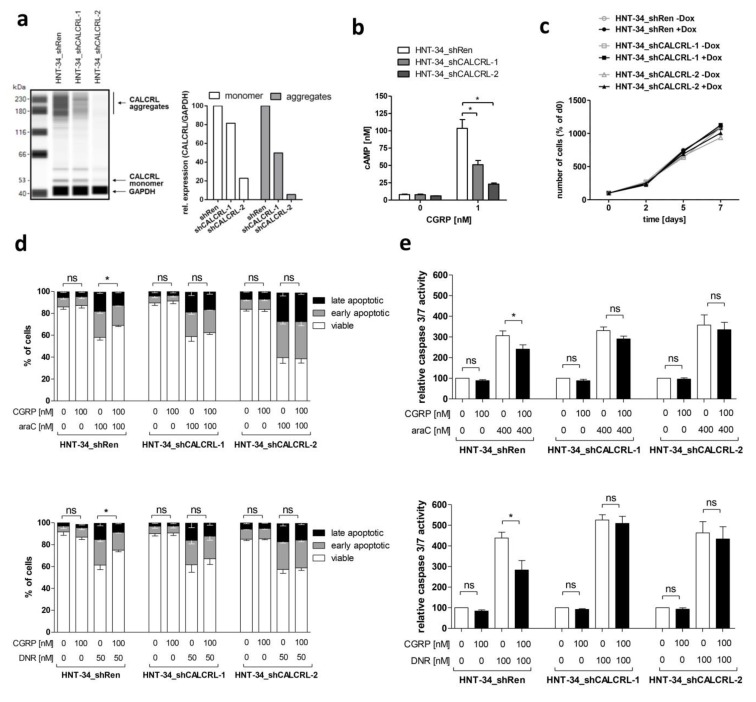
Genetic inhibition of *CALCRL* counteracts the CGRP-induced increase in chemotherapy resistance. *CALCRL* was knocked down in HNT-34 cells using lentivirus-borne, doxycycline inducible shRNAs. Unless indicated otherwise, experiments were performed after pre-incubation with and in the presence of doxycycline. (**a**) Expression of CALCRL in HNT-34_shRen, HNT-34_shCALCRL-1, and HNT-34_shCALCRL-2 was determined by capillary-based protein quantification analysis (Wes). Left panel, Wes plot; right panel, quantification. Protein aggregates are frequently observed with G-protein coupled receptors. (**b**) cAMP levels after stimulation of serum starved HNT-34_shRen, HNT-34_shCALCRL-1, and HNT-34_shCALCRL-2 with 0 or 1 nM CGRP for 2 min. Means + SEM from 3 biological replicates. * *p* < 0.05; paired Student’s two-tailed t-test. (**c**) Proliferation of HNT-34_shRen, HNT-34_shCALCRL-1, and HNT-34_shCALCRL-2 in the absence and presence of doxycycline. Means +/− SEM from 3 biological replicates (error bars do not exceed data point symbols in most cases). (**d**,**e**) *CALCRL* knock-down diminishes CGRP mediated protection from araC- and daunorubicin (DNR)-induced apoptosis. Cells were pre-incubated with or without 100 nM CGRP for 1 h prior to addition of the indicated concentrations of cytostatic drugs. Means +/− SEM from 3–4 biological replicates. ns, not significant, * *p* < 0.05, paired Student’s two-tailed *t*-test. (**d**) AnnexinV/DAPI assay. Significance indicators refer to the proportion of viable cells. (**e**) Caspase 3/7 assay.

To verify that the chemoprotective effect of CGRP was dependent on its cognate receptor CALCRL, HNT-34 derivative cell lines containing either of two different shRNAs against CALCRL (HNT-34_shCALCRL-1, HNT-34_shCALCRL-2) or an shRNA against Renilla luciferase as a control (HNT-34_shRen) were established. Cells were incubated with doxycycline to induce shRNA expression, and down-regulation of CALCRL in HNT-34_shCALCRL-1 and HNT-34_shCALCRL-2 compared to HNT-34_shRen was confirmed using capillary-based protein quantification analysis (Figure 3a; receptor aggregates are often observed with G-protein coupled receptors due to their high hydrophobicity). As expected, stimulation of HNT-34_shRen with CGRP increased intracellular cAMP levels, and this response was reduced in HNT-34_shCALCRL-1 and HNT-34_shCALCRL-2 (Figure 3b). Agreeing with the absence of a proliferative response to CGRP, induction of shRNA expression with doxycycline had no effect on cell proliferation (Figure 3c). However, knock-down of *CALCRL* diminished the protective effect of CGRP toward apoptosis induced by araC and daunorubicin, as determined through the AnnexinV/DAPI assay (Figure 3d, Figure S3), and corroborated through a caspase 3/7 activity assay (Figure 3e). These data indicate that CGRP promotes chemotherapy resistance in a *CALCRL* dependent, and therefore specific, manner.

### 2.4. Pharmacological Inhibition of the CGRP Receptor Counteracts the CGRP-Induced Increase in Chemotherapy Resistance in Human AML Cell Lines and Primary Samples

**Figure 4:**
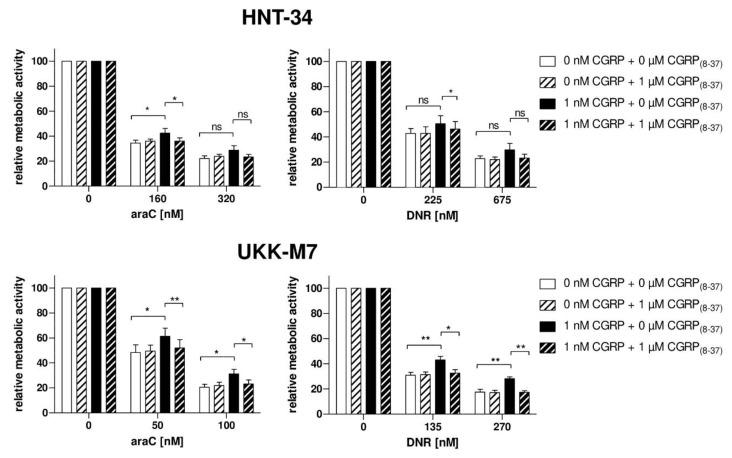
CGRP_(8–37)_ counteracts the CGRP-induced increase in chemotherapy resistance. HNT-34 and UKK-M7 cells were pre-incubated first with or without 1 µM CGRP_(8–37)_ for 15 min, next with or without 1 nM CGRP for 1 h, prior to addition of the indicated concentrations of araC or daunorubicin (DNR). CellTiter-Glo metabolic activity assay; means + SEM from 3 biological replicates. ns, not significant, * *p* < 0.05, ** *p* < 0.01, paired Student’s two-tailed t-test.

**Figure 5:**
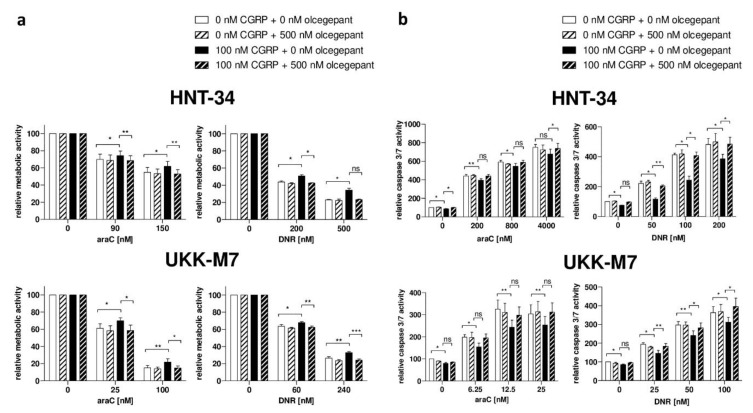
Olcegepant counteracts the CGRP-induced increase in chemotherapy resistance. HNT-34 and UKK-M7 cells were pre-incubated first with 500 nM olcegepant or an equivalent amount of solvent for 15 min, next with or without 100 nM CGRP for 1 h, prior to addition of the indicated concentrations of araC or daunorubicin (DNR). Means + SEM from 3–5 biological replicates. ns, not significant, * *p* < 0.05, ** *p* < 0.01, *** *p* < 0.001, paired Student’s two-tailed t-test. (**a**) CellTiter-Glo metabolic activity assay. (**b**) Caspase 3/7 assay.

To further confirm the specificity of the chemoprotective effect of CGRP, and to explore the potential of small molecules to counteract this activity, the N-terminally truncated, antagonistic peptide CGRP_(8–37)_ as well as the highly specific small molecule inhibitor olcegepant were used. In the absence of CGRP, CGRP_(8–37)_ had no effect on the sensitivity of HNT-34 and UKK-M7 cells to araC and daunorubicin, as measured through a metabolic activity assay (Figure 4). However, pre-incubation with an excess of CGRP_(8–37)_ restored cellular chemotherapy sensitivity in the presence of CGRP to levels comparable to those observed in its absence, and did so in both cell lines and for both drugs (Figure 4). Similar effects were observed with olcegepant both in the metabolic activity (Figure 5a) and the caspase 3/7 assay (Figure 5b).

**Figure 6:**
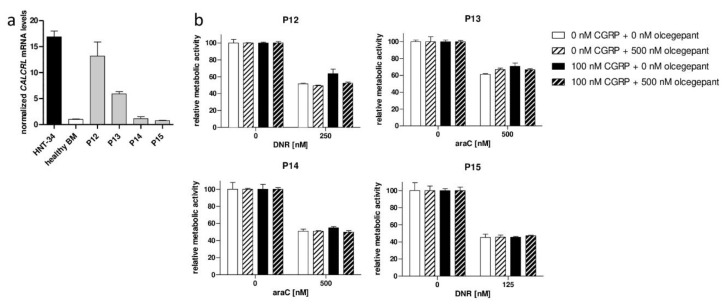
CGRP increases drug resistance of CALCRL positive primary AML cells and is counteracted by olcegepant. (**a**) Expression of *CALCRL* in HNT-34 cells, healthy control BM MNCs, and primary human AML samples (P12–P15) was determined by qRT-PCR. Normalization to β-2-microglobulin and calibration to the control BM sample was performed using the ΔΔC_T_ method. Means + SD from technical replicates. (**b**) CellTiter-Glo metabolic activity assay. Cells were pre-incubated first with 500 nM olcegepant or an equivalent amount of solvent for 15 min, next with or without 100 nM CGRP for 1 h, prior to addition of the indicated concentrations of araC or daunorubicin (DNR). Means + SD from technical replicates. Statistical tests were not performed because no biological replicates can be performed with patient samples, and statistical significance based on technical replicates is biologically meaningless.

The chemoprotective activity of CGRP and its abrogation by olcegepant were further confirmed using primary human AML samples. Compared to healthy BM MNCs, AML P12 and P13 expressed elevated, and AML P14 and P15 normal, levels of *CALCRL* (Figure 6a). Accordingly, the drug resistance of AML P12 and P13 was increased by CGRP, and this effect was abrogated by olcegepant, but very weak or no effects were observed for AML P14 and P15 (Figure 6b).

### 2.5. In Vivo Treatment with the CGRP Antagonist Olcegepant Increases Differentiation and Decreases Leukemic Burden and Stem Cell Properties in a Mouse Model of AML

**Figure 7:**
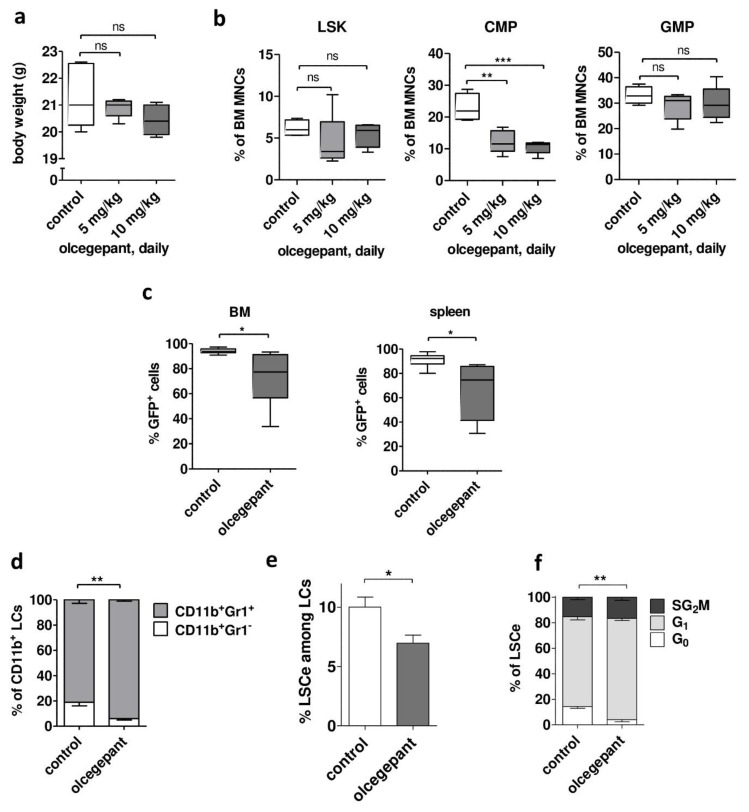
Olcegepant decreases leukemic burden and stem cell properties in a mouse model of AML. (**a**,**b**) Healthy C57BL/6 mice (4–5 per group) were treated with 0, 5, or 10 mg/kg olcegepant by daily *i.p.* injection from day 1–14 after a sublethal irradiation. ns, not significant, ** *p* < 0.01, *** *p* < 0.001, Student’s two-tailed t-test. (**a**) Total body weight at the end of treatment. (**b**) Relative abundance of LSK cells, CMPs, and GMPs among BM MNCs. (**c**–**f**) C57BL/6 mice were transplanted with LC^LSK_MA9^ and treated with 0 or 10 mg/kg olcegepant by daily *i.p.* injection from day 7–18 after transplantation. * *p* < 0.05, ** *p* < 0.01, Student’s two-tailed t-test. (**c**) Leukemic burden: proportion of Venus^+^ (leukemic) cells in bone marrow (BM) and spleen. n = 6/group. (**d**) Leukemic cell (LC) differentiation: proportion of Gr1^+^ cells among Venus^+^ CD11b^+^ BM cells. (**e**) Abundance of the LSC enriched Lin^−^ Sca1^−^ c-Kit^+^ CD34^+^ CD16/CD32^hi^ (LSCe) population among leukemic (Venus^+^) cells (LCs) in BM. (**f**) Cell cycle distribution of LSCe. Significance indicator refers to G_0_ only. (**d**–**f**) *n* = 5/group.

To begin to explore the possible utility of small-molecule CGRP antagonists for the treatment of AML, a mouse model was used in which AML is driven by *MLL-AF9* (*MA9*), a leukemogenic fusion gene recurrently found in human AML [48,49,50]. This model is one of the best characterized AML models, and one of the very few for which an immuno-phenotype characterizing a strongly LSC-enriched cell population (LSCe) has been described (Lin^−^ Sca1^−^ c-Kit^+^ CD34^+^ CD16/CD32^hi^ [49,51]). *Calcrl* and *Ramp1* were expressed both in BM from healthy C57BL/6 mice and in leukemic cells (LC^LSK_MA9^) from mice that had developed AML after transplantation with *MA9*-transduced LSK cells (Figure S4a). Olcegepant was developed for the treatment of migraines and exhibited a favorable toxicity profile in humans [1]. However, data for its maximal tolerability in the C57BL/6 strain and its administration via the *i.p.* route were not available in the literature. Therefore, healthy C57BL/6 mice were sub-lethally irradiated (5 Gy; to match the conditions employed in the AML model) and, starting on the next day, treated daily with 5 or 10 mg/kg olcegepant *i.p.* for two weeks. Mice were sacrificed at the end of the treatment period. Neither dose of olcegepant had a significant effect on total body weight (Figure 7a), liver or spleen weight (Figure S4b), white blood cell count (WBC), red blood cell count (RBC), or platelet counts (Figure S4c), or on the relative abundance of LSK cells or granulocyte macrophage progenitors (GMPs) in BM (Figure 7b). To contrast, olcegepant significantly reduced the proportion of common myeloid progenitors (CMPs) in BM (Figure 7b), confirming that hematopoietic cells in BM were exposed, and responsive, to CGRP. Nevertheless, the absence of an effect of olcegepant on all other measured parameters, including on the strongly hematopoietic stem cell enriched LSK population, suggested that its toxicity may be tolerable. Therefore, the impact of *in vivo* treatment with olcegepant on the emergence and properties of *MA9*-driven AML were investigated. C57BL/6 mice were sub-lethally irradiated (5 Gy) and transplanted with LC^LSK_MA9^. Starting on day 7 after transplantation, mice were treated with 10 mg/kg olcegepant or an equivalent amount of vehicle by daily *i.p.* injection for up to 12 days. Due to rapid disease progression, all mice were sacrificed between day 8 and 12 of treatment, and hematopoietic organs were collected and analyzed. Olcegepant had no effect on spleen weight, WBC, RBC, or platelet counts (Figure S4d,e). However, it significantly decreased the proportion of leukemic (Venus^+^) cells in BM and spleen (Figure 7c) and increased the proportion of the more mature Gr1^+^ cells among myeloid leukemic (CD11b^+^ Venus^+^) cells (Figure 7d, Figure S4f). Most importantly, olcegepant diminished LSC properties: it reduced both the abundance of LSCe and one of their key properties, namely, proliferative quiescence (Figure 7e,f, Figure S4g,h).

To summarize, olcegepant monotherapy decreased leukemic burden and increased differentiation in a mouse model of human AML. Furthermore, it decreased the abundance and quiescence of a cell population enriched for LSCs, which play a key role in therapy resistance.

## 3. Discussion

High *CALCRL* expression was an independent prognostic parameter for poor outcomes of AML in several publicly available data sets comprising patient populations with different genetic and age compositions (Table 1, Table S1). Similar results were recently reported by others [35,41], with some overlap and some divergence regarding the data sets used in each of these studies. Moreover, studies on paired samples showed that *CALCRL* was consistently up-regulated at relapse of AML (Figure 1a, and [34,35]), a disease stage characterized by increased chemotherapy resistance as compared to AML at diagnosis. *CALCRL* mRNA levels were higher in LSC enriched *versus* LSC depleted AML cell populations (Figure 1c), and genes co-expressed with *CALCRL* were significantly enriched for LSC signatures [41]. *CALCRL* also was expressed at higher levels in stem cell enriched CD34^+^ populations from AML patients as compared to those from healthy donors (Figure 1d). Together, these data raise the possibility that CALCRL may represent a novel therapeutic target in AML: its expression (i) correlates with chemotherapy resistance; (ii) is high in LSCs, the cell type that needs to be targeted for successful eradication of AML; and (iii) is increased in leukemic over normal hematopoietic stem cells, indicating the existence of a potential therapeutic window.

Even though two recent studies have investigated the prognostic role of *CALCRL* in AML [35,41], functional data were so far restricted to the observation that its knock-out reduced the clonogenic activity of human myeloid cell lines [41]. We show here that addition of CGRP to two receptor (CALCRL + RAMP1) positive human AML cell lines did not affect their proliferation in suspension culture, but increased their resistance to araC and daunorubicin, the drugs representing the mainstay of AML treatment. This effect was specific, because it was diminished by shRNA-mediated knock-down of *CALCRL* and abrogated by the CGRP antagonists CGRP_(8–37)_ and olcegepant. The impact of CGRP on drug resistance in AML may be underestimated in these cell line models, because araC increased the expression of the *CALCRL* and *RAMP1* mRNAs in patient samples (Figure 1b), but not in the cell lines (Figure S5). Nevertheless, we did not observe a bigger chemoprotective effect of CGRP on receptor positive primary AML samples than on the cell lines (Figure 2c and Figure 6b), possibly because in these short term *in vitro* assays the newly transcribed mRNAs had not yet given rise to substantially increased amounts of functional receptor at the cell surface. Since araC is administered for 7 days per treatment cycle in the clinical setting, it remains possible that in this context, receptor up-regulation contributes to resistance to and escape from therapy of a (probably small) sub-population of AML cells. Furthermore, irradiation, a genotoxic stressor like araC and daunorubicin, increased the expression of CGRP mRNA and protein in murine BM stromal cells [39]. Together, these data raise the intriguing possibility that AML chemotherapy may induce resistance against itself by augmenting signaling through the CGRP-CALCRL axis.

In a first attempt to identify downstream mediators of CGRP-CALCRL signaling in AML, the cytokine secretome of HNT-34 cells exposed to CGRP for various periods of time was determined (Figure S6). Among others, CGRP stimulated the secretion of IL-8, which also has been implicated in chemotherapy resistance in AML and may represent an alternative druggable target [52].

In an *MA9*-driven, *Calcrl*/*Ramp1* positive mouse model of AML, *in vivo* treatment with olcegepant increased the differentiation of leukemic cells and reduced leukemic burden as well as the abundance and quiescence of an LSC enriched cell population. These effects are particularly remarkable because *MA9* causes very aggressive AML in mice that nevertheless expressed *Calcrl* only at moderate levels, and because olcegepant has a ~200-fold lower affinity for the murine *versus* the human CGRP receptor [1]. Together with the cell line data, the results of this monotherapy experiment indicate that CGRP inhibition may display multiple anti-leukemic activities: like *all-trans* retinoic acid [53,54], the first targeted therapy developed for any subtype of AML, it induced maturation of myeloid leukemic cells. However, in contrast to retinoic acid, CGRP inhibition additionally increased chemotherapy sensitivity of AML in a dual manner: firstly, by augmenting drug responsiveness of receptor positive cells, and secondly, by decreasing the abundance of quiescent LSCs, an intrinsically more therapy resistant cell type (with the latter effect occurring independently of the presence of therapeutic drugs).

As expected, based on the favorable safety profiles of olcegepant and related compounds (gepants) in humans [1,5], a 2-week treatment with olcegepant displayed little if any toxicity in healthy mice (Figure 7a,b). Specifically, in the hematopoietic system, it did not affect spleen or liver weight, WBC, RBC, platelet counts, or the relative abundance of LSK cells or GMPs in BM, yet it did effect a decrease in the proportion of BM CMPs. Initially, the lack of an effect on stem cell enriched murine LSK cells might seem at odds with a report showing that stem cell enriched human CD34^+^ cells were CGRP responsive [38]. However, the two sets of experiments are hardly comparable since, in one case, cAMP levels were measured after *in vitro* exposure to CGRP, while in the other, LSK abundance was determined after *in vivo* treatment with olcegepant. Moreover, a prospectively isolated human CD34^+^ population does not equal an analytically defined murine LSK population. To contrast, our data agree well with the observation that receptor tachyphylaxis following a 2-week *in vivo* treatment with CGRP was associated with a relative decrease of CMPs but did not affect the proportions of a (not explicitly defined) HSC population or of other progenitor populations [39]. Interestingly, even though *in vivo* exposure to olcegepant or CGRP had no effect on the relative abundance of LSK cells (Figure 7b) or “HSCs” [39], respectively, *in vitro* CGRP treatment decreased HSC activity, as determined in a serial replating assay [39]. While this apparent discrepancy requires further scrutiny, the latter result suggests that antagonizing CGRP might positively affect HSC activity, and thus, recovery from chemotherapy. On the other hand, targeted deletion of *Ramp1* reduced normal hematopoiesis under certain stress conditions [39], indicating that the effects of CGRP-CALCRL inhibition on the residual normal hematopoiesis in an AML setting requires thorough investigation.

Our expression analyses identified the CGRP receptor as the primary CALCRL containing receptor in human AML. The *RAMP1* mRNA was amplified from all investigated patient samples, while *RAMP2* was amplified from none, and *RAMP3* only from 3/12. We were not able to identify an AML cell line expressing *RAMP3*; however, UKK-M7 cells expressed *RAMP2* in addition to *RAMP1* (Figure S7a). Correspondingly, they exhibited increased resistance toward cytostatic drugs not only in response to CGRP, but also to adrenomedullin (Figure S7b). Despite the discrepancy between primary samples and cell lines regarding the identity of the expressed coreceptor, these data raise the possibility that, in a small subgroup of patients, CALCRL may mediate chemotherapy resistance not only after stimulation by CGRP, but also by adrenomedullin. Theoretically, drugs inhibiting CALCRL signaling irrespective of ligand identity are conceivable; practically, it may be difficult to achieve the required specificity, and inhibiting adrenomedullin signaling may be associated with a wider spectrum of on-target adverse events. Therefore, as with all targeted drugs, it will be important to precisely define which patients may benefit from a possible therapy containing CALCRL antagonists.

Intense efforts are made to develop drugs targeting the CGRP-CALCRL axis, primarily because of its causative role in migraines, a frequent condition affecting 18% of women and 8% of men [1,5]. Olcegepant was the first high-affinity small molecule CGRP antagonist [1]. Despite promising initial results, the clinical development of gepants of this generation was discontinued due to liver toxicity (telcagepant) or the fact that they were not orally bioavailable (olcegepant) [1,5]. Newer gepants with improved specificity did not cause any significant cardiac or hepatic adverse events [5]; ubrogepant was recently approved by the FDA (https://www.drugs.com/history/ubrogepant.html) and approval of rimegepant is pending (https://www.biohavenpharma.com/science-pipeline/cgrp/rimegepant). Furthermore, several monoclonal antibodies targeting the CGRP-CALCRL interaction, which exhibit higher target specificity, fewer drug–drug interactions, and longer circulating half-lifes than gepants, have been approved for clinical use [5]. Thus, a number of clinically fully developed agents are available that can be explored for their abilities to reduce chemotherapy resistance and stemness in CALCRL positive AML. Additional issues to be addressed are potential molecular escape mechanisms, possible adverse interactions between CGRP antagonists and cytotoxic therapy, and the optimal timing and dosing of these two classes of drugs.

## 4. Materials and Methods

### 4.1. Ethics Statement

Experiments with primary human AML samples were approved by the Ethics Committee of the Medical University of Vienna (EK 179/2011, issued on 15 Mar 2011, and EK 1394/2019, issued on 13 June 2019) and conducted in accordance with the declaration of Helsinki. Informed consent was obtained prior to establishment of the cell line UKK-M7, and all pertinent ethical regulations in place at that time at the University of Cologne were observed. Animal experiments were approved by the Animal Ethics Committee of the Medical University of Vienna and the Austrian Federal Ministry of Education, Science, and Research (BMWFW-66.009/0309-WF/V/3b/2015, issued on 3 Nov 2015). Austrian and Federation of European Laboratory Animal Science Associations guidelines to minimize animal distress and suffering were followed.

### 4.2. Patient Samples and Healthy Controls

Primary samples from patients with AML were provided by the Medical University of Graz, Austria (11 sample pairs from diagnosis and relapse, used for expression analyses) and by the Hanusch Hospital, Vienna, Austria (4 diagnostic samples, used for functional assays). Nine of 11 sample pairs subjected to expression analysis had been used for genome-wide gene expression profiling in a previous report [34]; four of these (P2, P3, P4, P6) were used both for initial confirmation of the expression changes between diagnosis and relapse and to investigate the effect of araC on *CALCRL* expression. Patients’ clinical characteristics are summarized in Table S2. BM MNCs and CD34^+^ hematopoietic stem and progenitor cells from 2 healthy donors each were purchased from Lonza (Basel, Switzerland).

Concerning expression analyses, cells were prepared and, where indicated, treated with araC (4 µM for 48 h) as described previously [34,55]. Regarding metabolic activity assays, vitally frozen AML cells were thawed, washed with RPMI 1640 medium (Invitrogen, CA, USA) supplemented with 10% fetal bovine serum (FBS; Invitrogen), 1% Penicillin/Streptomycin/Glutamine (Invitrogen), and 5 µg/mL DNase (Sigma–Aldrich, Missouri, USA), and incubated for 60 min at 37 °C and 5% CO_2_ with RPMI 1640 medium containing 10% FBS, 1% Penicillin/Streptomycin/Glutamine, and 50 µg/mL DNase to prevent cell clumping. Cells were washed with PBS (Invitrogen) and cultured in RPMI 1640 medium containing 10% FBS, 1% Penicillin/Streptomycin/Glutamine, and 100 ng/mL each of SCF (Peprotech, Hamburg, Germany), IL-3 (Peprotech), and G-CSF (Peprotech). After overnight recovery, cells were seeded to a final density of 100/µL. Five hundred nM olcegepant (MedChemExpress, Monmouth Junction, NJ, USA; 500 µM stock in DMSO (AppliChem, Darmstadt, Germany)), 100 nM CGRP (Phoenix Pharmaceuticals, Burlingame, CA, USA; 100 µM stock in ultra-pure H_2_O (Gibco, Thermo Fisher Scientific, Massachusetts, USA)), and cytostatic drugs were added sequentially, with pre-incubation times of 15 and 60 min for olcegepant and CGRP, respectively, and including appropriate negative controls. araC and daunorubicin (provided by the dispensary of the General Hospital of Vienna) were used at patient-specific IC_50_ concentrations (500 nM araC for P13 and P14; 125 and 250 nM daunorubicin for P15 and P12, respectively). Twenty-four hours after the addition of cytostatic drugs, metabolic activity was determined using the CellTiter-Glo Assay (Promega, Wisconsin, USA) according to the manufacturer’s instructions. Measurements were performed on a Varioskan LUX plate reader with SkanIt Software for Microplate Readers RE, Version 5.0.0.42. (Thermo Fisher Scientific, Massachusetts, USA).

### 4.3. Establishment of a New AML Cell Line, UKK-M7

A 55 year old male patient with a brief antecedent history of hematological malignancy (T-cell non-Hodgkin lymphoma successfully treated with cyclophosphamide, hydroxydaunorubicin, vincristine (Oncovin^®^), and prednisone (CHOP)) was diagnosed in July 1994 with AML M7 at the University Hospital of Cologne. His blasts were positive for CD41 (98%), CD42 (82%), CD61 (95%), CD33 (90%), CD34 (60%), CD36 (39%), and HLA-DR (93%). Despite intensive chemotherapy (thioguanine, araC, and daunorubicin (TAD); high dose araC and mitoxantrone (HAM)), blast persistence was diagnosed in September 1994. Two weeks later, after obtaining informed consent, peripheral blood was collected and used to establish the cell line UKK-M7. Further intensive therapy was unsuccessful, and the patient died of refractory AML in November 1994.

The mononuclear fraction of the blood sample was enriched by Ficoll density centrifugation and cultured in RPMI 1640 medium supplemented with 10% FCS and 5 ng/mL each GM-CSF and G-CSF (both from R and D Systems, Minneapolis, MN, USA). After 6 months, a cytokine independent sub-line with the immuno-phenotype CD34^+^, CD38^+^, CD33^+^, HLA DR^+^ and a complex karyotype, 45, XY, t(1;3)(p13-21;q12), del(3)(q?21), der(6)del(6)(q21)t(6;7)(q21;?),-7, ?(dic8)add(8)(?q24), add(12)(p13), der(17)t(8;17)(?q24;p12-13), which resembled that present in the patient’s blasts, was isolated. Further details are available upon request.

### 4.4. Cell Culture and Retroviral Transductions

Human UKK-M7 cells, as well as HNT-34 cells [47] (kindly provided by the laboratory of origin) and their derivatives were cultured in RPMI 1640 medium supplemented with 10% FBS and 1% Penicillin/Streptomycin (Sigma–Aldrich). All experiments were performed using logarithmically growing cells (2.5–8 × 10^5^ cells/mL). The packaging cell line Phoenix-GP was cultivated in DMEM (Invitrogen), 10% FBS, and 1% Penicillin/Streptomycin. MycoAlert mycoplasma detection kit (Lonza, Basel, Switzerland) was utilized regularly to demonstrate that cell lines were free of mycoplasma contamination.

Two different shRNAs against *CALCRL* (shCALCRL-1, 5′-GCAGTGTTTGTTTAAATGTAA-3′, and shCALCRL-2, 5′-CAGGCTATTCAAGAAATATAA-3′) were transferred from LPE_shCALCRL_4409S and LPE_shCALCRL_3188s (Mirimus, Brooklyn, NY, USA), respectively, into LT3REVIR, a lentiviral vector allowing for doxycycline inducible expression of its shRNA inserts [56]. LT3REVIR_Ren713, containing an shRNA against the Renilla luciferase gene (shRen) and kindly provided by Dr. Johannes Zuber, IMP, Vienna, Austria, was used as a control. shRNA vectors were transiently transfected into Phoenix-GP cells, along with packaging plasmids psPAX2 and pMD2.G, using a standard calcium chloride protocol. Forty-eight hours later, virus-containing supernatants were harvested and filtered (0.45 µm pore size). After addition of polybrene to a final concentration of 4 μg/mL, lentiviral supernatants were spinoculated onto HNT-34 cells at 1300 rpm and 32 °C for 60 min. Spinoculation was repeated with fresh lentiviral supernatants after 1 and 2 days. Three days after the last transduction cycle, cells were sorted for Venus positivity on an Astrios (Beckman Coulter, CA, USA). Sorting for Venus was repeated twice, followed by a sort for the doxycycline inducible marker dsRed after incubation with 2 µg/mL doxycycline (MP Biomedicals, Fisher Scientific, New Hampshire, USA) for 8 days, yielding the cell lines HNT-34_shCALCRL-1, HNT-34_shCALCRL-2, and HNT-34_shRen. To induce shRNA expression and allow for an optimal knock-down effect, HNT-34 derivative cell lines were treated with 2 µg/mL doxycycline for 4–8 days prior to performing experiments.

### 4.5. RNA Isolation, Reverse Transcription, and qRT-PCR

Total RNA was extracted using TRIzol and reverse transcribed using M-MLV reverse transcriptase and random hexamer primers (all from Life Technologies, CA, USA). Concerning human samples, qRT-PCR was performed using TaqMan Gene Expression Master Mix and TaqMan probes (*CALCRL*: Hs00907738_m1, *RAMP1*: Hs00195288_m1, *RAMP2*: Hs01594524_m1, *RAMP3*: Hs00389131_m1, and h*β*-2-microglobulin: Hs99999907_m1, Thermo Fisher). Regarding mouse samples, GoTaq qRT-PCR master mix (Promega, Fitchburg, WI, USA) was used together with primers *Calcrl*-fwd, 5′-GCAGCAGCTACCTAGCTTGAA-3′, *Calcrl*-rev, 5′-TTCACGCCTTCTTCCGACTC-3′, *Ramp1*-fwd, 5′-GGGAAGACGCTATGGTGTGA-3′, *Ramp1*-rev, 5′-TTCCGGATTGGGCCAGAAAC-3′, m*β*-2-microglobulin-fwd, 5′-CCTTCAGCAAGGACTGGTCT-3′, and m*β*-2-microglobulin-rev, 5′-TGTCTCGATCCCAGTAGACG-3′. All assays were performed in triplicate on a Step One Plus Real Time PCR system (Life Technologies), and expression of the genes of interest was normalized to the expression of *β*-2-microglobulin and to a calibrator sample using the ΔΔC_T_ method [57].

### 4.6. Capillary-Based Protein Quantification Analysis (Wes)

Protein extracts were prepared in HEPEX lysis buffer (20 mM Hepes pH 7.4, 100 mM NaCl, 5 mM EDTA, 1 mM Na_3_VO_4_, 30 mM NaF, 5% Glycerol, 0.1% SDS, 1% TritonX100, 1 mM Glycerophosphate, 1 mM DTT, 2% PMSF, 10 mM p-Nitrophenylphosphate) supplemented with protease-inhibitor cocktail (Roche, Basel, Switzerland #11873580001) and phosphatase inhibitor cocktails 1, 2 and 3 (Sigma–Aldrich). Capillary-based protein quantification analysis was performed using standard pack reagents (Protein Simple, CA, USA; Wes™) according to the manufacturer’s protocol (https://www.proteinsimple.com/documents/Analyzing_Integral_Membrane_Proteins_with_Simple_Western_RevA.pdf). Briefly, protein extracts were diluted in a 1/100 pre-diluted sample buffer (ProteinSimple, 10× Sample Buffer) to obtain a 0.2 mg/mL protein solution. A 5× fluorescent master mix was added to the samples (1/5 dilution) and proteins were denatured for 5 min at 95 °C. The primary antibodies for CALCRL (CRLR; ab173562, Abcam, Cambridge, UK) and for GAPDH (ab9485, Abcam) were diluted 1:30 and 1:1000, respectively, in Antibody Diluent 2. Chemiluminescence substrates (luminol-S and peroxide) were mixed in equal amounts. Biotinylated size standard, protein samples, Wes antibody diluent 2, primary antibody solutions, anti-rabbit secondary antibody solution (provided with the kit), luminol-peroxide mixture, and wash buffer were filled into 12–230 kDa Wes Separation Modules, as indicated in the manufacturer’s protocol included with the Wes Separation Modules. Plates as well as Wes 25-Capillary Cartridges were placed into a Wes device (Simple Westerns™) and run for 3 h. Quantification was done utilizing the integrated Compass software, version 4.0 (ProteinSimple, San Jose, CA, USA). Normalization was based on GAPDH protein levels.

### 4.7. Metabolic Activity and Apoptosis Assays

Logarithmically growing human AML cell lines (HNT-34, HNT-34 derivatives, and UKK-M7) were diluted to a final density of 100 cells/µL. CGRP antagonists, CGRP, and cytostatic drugs were added sequentially, with pre-incubation times of 15 and 60 min for the antagonists and for CGRP, respectively. CGRP_(8–37)_ (Phoenix Pharmaceuticals, 320 µM stock in ultra-pure H_2_O) was used at a final concentration of 1 µM. Olcegepant was used at a final concentration of 500 nM, and an equivalent amount of DMSO was added to negative controls. CGRP was used at a final concentration of 1 nM in experiments using CGRP_(8–37)_ as antagonist and in cAMP assays, or of 100 nM in all other experiments. araC and daunorubicin (provided by the dispensary of the Vienna General Hospital) were used at the final concentrations indicated in the respective figures; treatment with araC was for 48 h, and that with daunorubicin for 21–24 h.

Metabolic activity (as a proxy for viability) and caspase 3/7 activity were determined using the CellTiter-Glo Assay (Promega) and Caspase-Glo 3/7 Assay (Promega), respectively. The manufacturer’s instructions were followed, except in the case of CellTiter-Glo, the amount of lysis/luminescence reagent was reduced to 1/5, which did not affect assay results as determined in pilot experiments. Measurements were performed on a Varioskan LUX plate reader with SkanIt Software for Microplate Readers RE, Version 5.0.0.42. (Thermo Fisher Scientific) or on a BertholdTech TriStar plate reader (Berthold Technologies, Bad Wildbad, Germany) with MikroWin software, Version 4.41.

For the AnnexinV/DAPI assay, treated cells were washed with PBS, resuspended in AnnexinV binding buffer (10 mM Hepes pH 7.4, 140 mM NaCl, 2.5 mM CaCl_2_), and incubated with 2.5 µL AnnexinV-APC (BD Pharmingen, Franklin Lakes, NJ, USA) and 0.1 µL 4′,6-diamidino-2-phenylindole (DAPI, 1 mg/mL, BioLegend) in a total volume of 100 µL for 15 min at room temperature in the dark. Analyses were performed on an LSRFortessa (Becton Dickinson, New Jersey, USA) using FACS Diva software. Double negative cells were considered viable, AnnexinV positive cells early apoptotic, and double positive cells late apoptotic.

### 4.8. Measurement of cAMP Levels

HNT-34_shRen, HNT-34_shCALCRL-1, and HNT-34_shCALCRL-2 grown in the presence of doxycycline were serum starved overnight. Cells were seeded at 1000 cells/µL in PBS supplemented with 0.9 mM CaCl_2_, 0.49 mM MgCl_2_, and 0.1 mM 3-isobutyl-1-methylxanthine (IBMX, Sigma, 100 mM stock in DMSO), and stimulated with 0 or 1 nM CGRP for 2 min at 37 °C. cAMP levels were quantified using the HitHunter^®^ cAMP Assay for Small Molecules (DiscoverX). Luminescence signal was measured on a Varioskan LUX plate reader with SkanIt Software for Microplate Readers RE, Version 5.0.0.42. (Thermo Fisher Scientific) 5 h after the addition of the last reagent.

### 4.9. Mouse Model

To test the potential toxicity of olcegepant under conditions resembling those used later for the AML model, 6–8 week old female C57BL/6 mice (Department of Laboratory Animal Science and Genetics, Himberg, Austria) were sub-lethally irradiated (5 Gy). Starting on the next day, 0, 5, or 10 mg/kg olcegepant were injected intraperitoneally (*i.p.*) every 24 h for 2 weeks. These concentrations were chosen based on preclinical migraines models [58,59,60] and using established protocols to convert compound concentrations from rats to mice [61]. However, to account for different routes of administration, intravenous in the reference experiments [58,59,60] *versus* *i.p.* in our model, the mouse-adapted concentration was over-titrated 2–4 fold. At the end of the treatment period, mice were sacrificed, and blood cell counts were determined using a hematology analyzer Sysmex XN-350 (Sysmex, Kobe, Japan). The abundance of LSK cells, CMPs (Lin^−^ Sca-1^−^ c-Kit^+^ CD34^+^ CD16/CD32^low^ [62]), and GMPs (Lin^−^ Sca1^−^ c-Kit^+^ CD34^+^ CD16/CD32^hi^ [62]) was determined on an LSRFortessa (Becton Dickinson) after staining with the respective antibodies (Table 2).

C57BL/6 mice with *MA9*-driven AML [49] were generated by transplantation with pMSCV_MA9_IRES_Venus [63] transduced LSK cells as previously described [50]. For the *in vivo* treatment experiment, 6–8 week old female C57BL/6 mice were sub-lethally irradiated (5 Gy). On the next day, they were anesthesized by *i.p.* injection of 100 µl Ketasol/Rompun solution (18.5 mg/mL Ketasol (AniMedica, Senden, Germany), 1.5 mg/mL Rompun (Bayer, Leverkusen, Germany), 0.9% Sodium Chloride (Braun, Kronberg, Germany)), and transplanted retro-orbitally with leukemic cells (LC^LSK_MA9^, 5000/mouse) obtained from mice with established *MA9*-driven AML. From day 7 to day 18 after transplantation, 0 or 10 mg/kg olcegepant was administered *i.p*. every 24 h. Due to rapid disease progression in both treatment groups, all mice were sacrificed between day 14 and 18 after transplantation. Blood parameters were determined as described above. In addition, leukemic burden (proportion of Venus^+^ cells) in spleen and BM was assessed. Differentiation of myeloid leukemic cells (reflected by the proportion of Gr1^+^ cells among CD11b^+^ Venus^+^ cells) and the proportion of LSC enriched cells (LSCe; Venus^+^, Lin^−^ Sca1^−^ c-Kit^+^ CD34^+^ CD16/CD32^hi^ cells [49,51]) among leukemic cells were determined by flow cytometric analysis of BM cells stained with the respective antibodies (Table 2).

To query the proportion of quiescent LSCe, BM cells were stained for LSCe surface markers, fixed and permeabilized in Cytofix/Cytoperm (BD Biosciences), stained with Ki-67 antibody (Table 2) and DAPI, and subjected to flow cytometry. The cut-off for Ki-67 positivity was determined using an isotype control antibody. Among cells in the LSCe gate, Ki-67 negative cells with a 2N DNA content were considered to be in G_0_. Flow cytometry was performed on an LSRFortessa (Becton Dickinson), and data were analyzed with FlowJo software, Version 10 (Tree Star, OR, USA).

### 4.10. Statistical and Bioinformatics Methods

Concerning experiments involving cell lines or mice, at least 3 biological replicates were performed, and data were represented as means +/− SEM. The two-tailed t-test was used to test for statistical significance. Regarding experiments with patient samples, technical replicates were performed and results were represented as means +/− SD. For group comparisons of gene expression data, variants of the t-test were used as follows: BM MNCs *versus* BM CD34^+^ cells, two-tailed t-test; diagnosis *versus* relapse, paired two-tailed t-test; diagnosis or relapse *versus* BM MNCs, Welch’s t-test for unequal sample sizes; untreated *versus* araC treated AML samples, 1-sample t-test.

To compare the expression of *CALCRL* and *RAMP1* between different healthy and leukemic cell populations, the data sets GSE30377 [42], GSE76008 [22], and GSE30029 [43] were used. Statistical significance was assessed using the lmFit function of the R package Limma, which implements multiplicity correction for all probe sets present on an array. The prognostic significance of *CALCRL* and *RAMP1* was interrogated in data sets GSE12417 [18], GSE6891 [44], GSE37642 [45], GSE71014 [46], and TCGA [19]. Known prognostic parameters provided with the respective data sets were first tested in univariable analyses; those that resulted as significant were included in the multivariable models. Regarding Kaplan–Meier analyses, optimal cut-offs for each data set were determined using maximally selected rank statistics (R package *maxstat*) as previously described [50]. Statistical significance was assessed through the log-rank test. Survival analyses were performed using the R packages survival and survminer.

## Figures and Tables

**Table 1:** Prognostic significance of *CALCRL* in publicly available AML gene expression data sets.

	*CALCRL*, Univariable			*CALCRL*, Multivariable		
Accession Number	HR	95% CI	*p*-Value	HR	95% CI	*p*-Value
GSE12417, cohort 1	2.13	1.38–3.3	0.0007	1.97	1.27–3.07	0.002
GSE12417, cohort 2	2.5	1.4–4.5	0.002	1.76	0.89–3.48	0.105
GSE6891, cohort 1	2.1	1.49–2.91	1.7 × 10^−5^	1.58	1.1–2.3	0.012
GSE6891, cohort 2	2.65	1.8–3.9	6.1 × 10^−7^	2.3	1.5–3.4	6.4 × 10^−5^
GSE37642	1.78	1.34–2.35	5.6 × 10^−5^	1.64	1.2–2.2	0.0007
GSE71014	4.38	2.24–8.56	1.5 × 10^−5^		n.a.	
TCGA_LAML	2.09	1.26–3.46	0.0042	1.63	0.91–2.74	0.066

HR, hazard ratio; CI, confidence interval; n.a., not applicable (no information on other prognostic parameters provided in data set). Characteristics of the data sets are summarized in Table S1.

**Table 2:** Antibodies used for flow cytometry.

Target	Clone	Fluorophor	Company	Dilution
Mouse Gr-1	RB6-8C5	AF700	Biolegend	1:100
Mouse Gr-1	RB6-8C5	APC	Biolegend	1:100
Mouse CD11b	M1/70	AF700	Biolegend	1:100
Mouse CD3	17A2	AF700	Biolegend	1:100
Mouse B220	RA3-6B22	AF700	Biolegend	1:100
Mouse Ter119	TER119	AF700	Biolegend	1:100
Mouse c-Kit	2B8	APC-Cy7	Biolegend	1:50
Mouse Sca-1	D7	PerCP/Cy5.5	Biolegend	1:50
Mouse CD34	MEC14.7	PE/Cy5.5	Biolegend	1:50
Mouse CD16/CD32	93	PE/Cy7	eBioscience	1:50
Mouse Ki-67	16A8	APC	Biolegend	1:50

AF700 labelled Gr-1, CD11b, CD3, B220, and Ter119 antibodies were combined to define Lin^−^ cells.

## Supplementary Materials

Supplementary materials can be found at https://www.mdpi.com/1422-0067/20/23/5826/s1.

## Abbreviations

ADM	adrenomedullin
AML	acute myeloid leukemia
araC	cytosine arabinoside
BM	bone marrow
BM MNC	bone marrow mononuclear cell
CALCRL	calcitonin receptor-like receptor
cAMP	cyclic adenosine monophosphate
CGRP	calcitonin gene-related peptide
CGRP_(8–37)_	truncated CGRP peptide missing amino acids 1–7
CMP	common myeloid progenitor
DAPI	4′,6-diamidino-2-phenylindole
DNR	daunorubicin
FBS	fetal bovine serum
GAPDH	glyceraldehyde 3-phosphate dehydrogenase
GMP	granulocyte/monocyte progenitor
HSC	hematopoietic stem cell
*i.p.*	intra-peritoneal
LC	leukemic cell
LSC	leukemic stem cell
LSCe	leukemic stem cell enriched
LSK	Lin^−^ Sca-1^+^ Kit^+^
M-MLV	moloney murine leukemia virus
MA9	MLL-AF9
MNC	mononuclear cell
PBS	phosphate-buffered saline
PCR	polymerase chain reaction
PLT	platelet
qRT-PCR	quantitative real time polymerase chain reaction
RAMP	receptor activity modifying protein
RBC	red blood cell
SD	standard deviation
SEM	standard error of the mean
shRNA	short hairpin RNA
TCGA	The Cancer Genome Atlas
WBC	white blood cell
Wes	capillary-based protein quantification analysis
