# Supplementary material for: CGRP Signaling via CALCRL Increases Chemotherapy Resistance and Stem Cell Properties in Acute Myeloid Leukemia
Source: Int J Mol Sci. 2019 Nov 20;20(23):5826. doi: 10.3390/ijms20235826 (PMC6928760; doi:10.3390/ijms20235826)
Supplement: Supplementary file 1 [file ijms-20-05826-s001.zip › ijms-616043-supplementary/Gluexam_Supplementary Material.docx]

**LEGENDS TO SUPPLEMENTARY FIGURES**

**Figure S1: Expression of *RAMP3*, and prognostic significance of *CALCRL* in AML.** (**a**) Expression of *RAMP3* in paired samples from diagnosis (Dx) and relapse (Rel) of AML was determined by qRT-PCR. Normalization to *β-2-microglobulin* and calibration to sample C4 was performed using the ΔΔC_T_ method. C1-C4, healthy control samples 1-4; BM, bone marrow; MNC, mononuclear cells; P1-P6, patients 1-6. Means + SD from technical replicates. (**b**) *CALCRL* mRNA levels in HSCs, progenitors (prog), and lineage marker positive (lin+) cells from healthy donors contained in GSE30377. FDR was determined using lmFit. (**c**) Kaplan-Meier curves showing the association between high *CALCRL* expression and poor outcome in AML data sets GSE12417, GSE6891, GSE37642, GSE71014, and TCGA. Optimal cut-offs for each data set were determined using maximally selected rank statistics. Significance was calculated using the log-rank test.

**Figure S2: CGRP increases the resistance of receptor positive human AML cell lines to drugs used in the treatment of AML.** HNT-34 and UKK-M7 cells were pre-incubated with or without 100 nM CGRP for 1 h prior to addition of the indicated concentrations of araC, and subjected to an AnnexinV/DAPI assay. Representative experiments.

**Figure S3:** **Genetic inhibition of *CALCRL* counteracts the CGRP-induced increase in chemotherapy resistance.** HNT-34_shRen, HNT-34_shCALCRL-1, and HNT-34_shCALCRL-2 were pre-incubated with or without 100 nM CGRP for 1 h prior to addition of 100 nM araC. AnnexinV/DAPI assay, representative experiment.

**Figure S4: Effects of *in vivo* treatment with olcegepant on healthy mice and in an *MA9*-driven mouse model of AML**. (**a**) Expression of *Calcrl* and *Ramp1* in healthy murine bone marrow (BM) cells (n = 3) and in leukemic cells from an *MA9*-driven mouse model of AML (LC^LSK_MA9^; n = 4) was determined by qRT-PCR. Normalization to m*β-2-microglobulin* was performed using the ΔΔC_T_ method. *, p<0.05, Student's two-tailed t-test. (**b, c**) Healthy C57BL/6 mice were treated with 0, 5, or 10 mg/kg olcegepant by daily *i.p.* injection from day 1-14 after a sublethal irradiation. ns, not significant; Student's two-tailed t-test. (**b**) Spleen and liver weight. (**c**) WBC, RBC, and platelet counts. (**d-h**) C57BL/6 mice were transplanted with LC^LSK_MA9^ and treated with 0 or 10 mg/kg olcegepant by daily *i.p.* injection from day 7-18 after transplantation. ns, not significant; Student's two-tailed t-test. (**d**) Spleen weight. (**e**) WBC, RBC, and platelet counts in peripheral blood. (**f**) Leukemic cell differentiation: proportion of Gr1^+^ cells among Venus^+^ CD11b^+^ BM cells, representative experiment. (**g**) Abundance of an LSC enriched (LSCe) population among leukemic (Venus^+^) cells in BM, representative experiment. (**h**) Cell cycle distribution of LSCe, representative experiment.

**Figure S5: araC does not up-regulate *CALCRL* or *RAMP1* expression in human AML cell lines.**

HNT-34 and UKK-M7 cells were treated with the indicated concentrations of araC for 2 days, and *CALCRL* and *RAMP1* mRNA levels were determined by qRT-PCR. Normalization to *β-2-microglobulin* was performed using the ΔΔC_T_ method. Means + SEM from 3 biological replicates. ns, not significant, **, p<0.01; Student's two-tailed t-test.

**Figure S6: CGRP alters the cytokine secretome of HNT-34 cells**

HNT-34 cells were seeded at a concentration of 500/µl, and treated or not with 100 nM CGRP for 1, 16, 24, or 48 h. After incubation, cells were removed from media via two centrifugation steps, and supernatants were stored at -80°C until analysis. The Bio-Rad Bio-Plex Pro Human Chemokine Panel, 40-Plex (#171AK99MR2) was used according to the manufacturer's protocol (http://www.bio-rad.com/webroot/web/pdf/lsr/literature/10031990.pdf). Means + SEM from 3 biological replicates. n.s., not significant; *, p<0.05; **, p<0.01; ***, p<0.001; Student's two-tailed t-test.

**Figure S7: UKK-M7 cells express RAMP2, and their resistance to chemotherapy is increased by adrenomedullin.** (**a**) *RAMP2* mRNA levels in HNT-34 and UKK-M7 cells were determined by qRT-PCR. Normalization to *β-2-microglobulin* was performed using the ΔΔC_T_ method. Means + SD from technical replicates. n.d., not detectable. (**b**) CellTiter-Glo metabolic activity assay. Cells were pre-incubated with or without 100 nM adrenomedullin for 1 h prior to addition of the indicated concentrations of cytostatic drugs. Means + SEM from 3 biological replicates. ns, not significant, *, p<0.05, paired Student's two-tailed t-test.

**SUPPLEMENTARY TABLES**

Table S1: Summary of publicly available AML gene expression data sets used to test prognostic significance of *CALCRL* and *RAMP1*, and Cox regression analyses

Table S2: Clinical characteristics of patients with AML
