# Supplementary figures and images for: CGRP Signaling via CALCRL Increases Chemotherapy Resistance and Stem Cell Properties in Acute Myeloid Leukemia
Source: Int J Mol Sci. 2019 Nov 20;20(23):5826. doi: 10.3390/ijms20235826 (PMC6928760; doi:10.3390/ijms20235826)

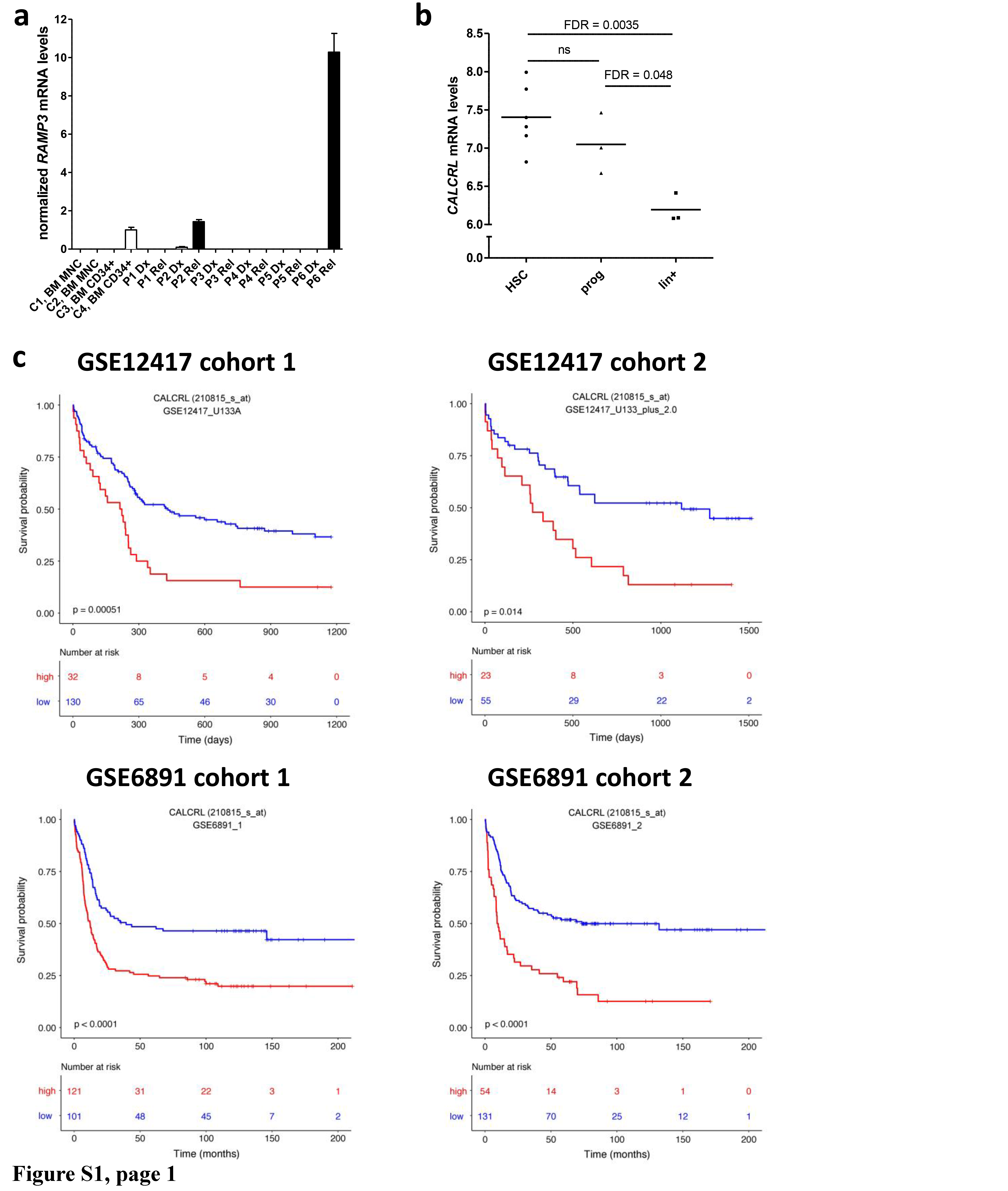

Supplement: Supplementary file 1 [file ijms-20-05826-s001.zip › ijms-616043-supplementary/FigS1_s1.tif]

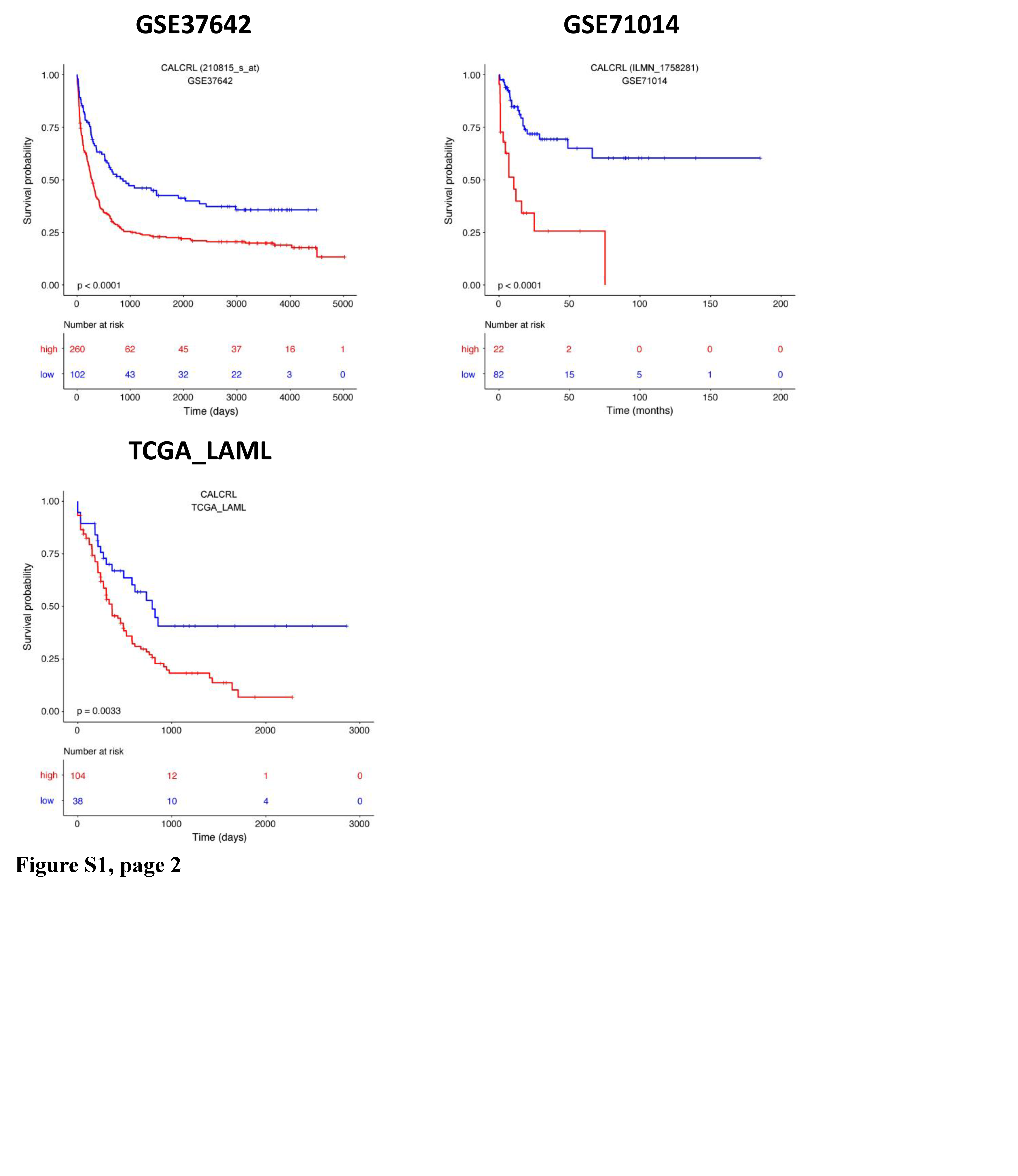

Supplement: Supplementary file 1 [file ijms-20-05826-s001.zip › ijms-616043-supplementary/FigS1_s2.tif]

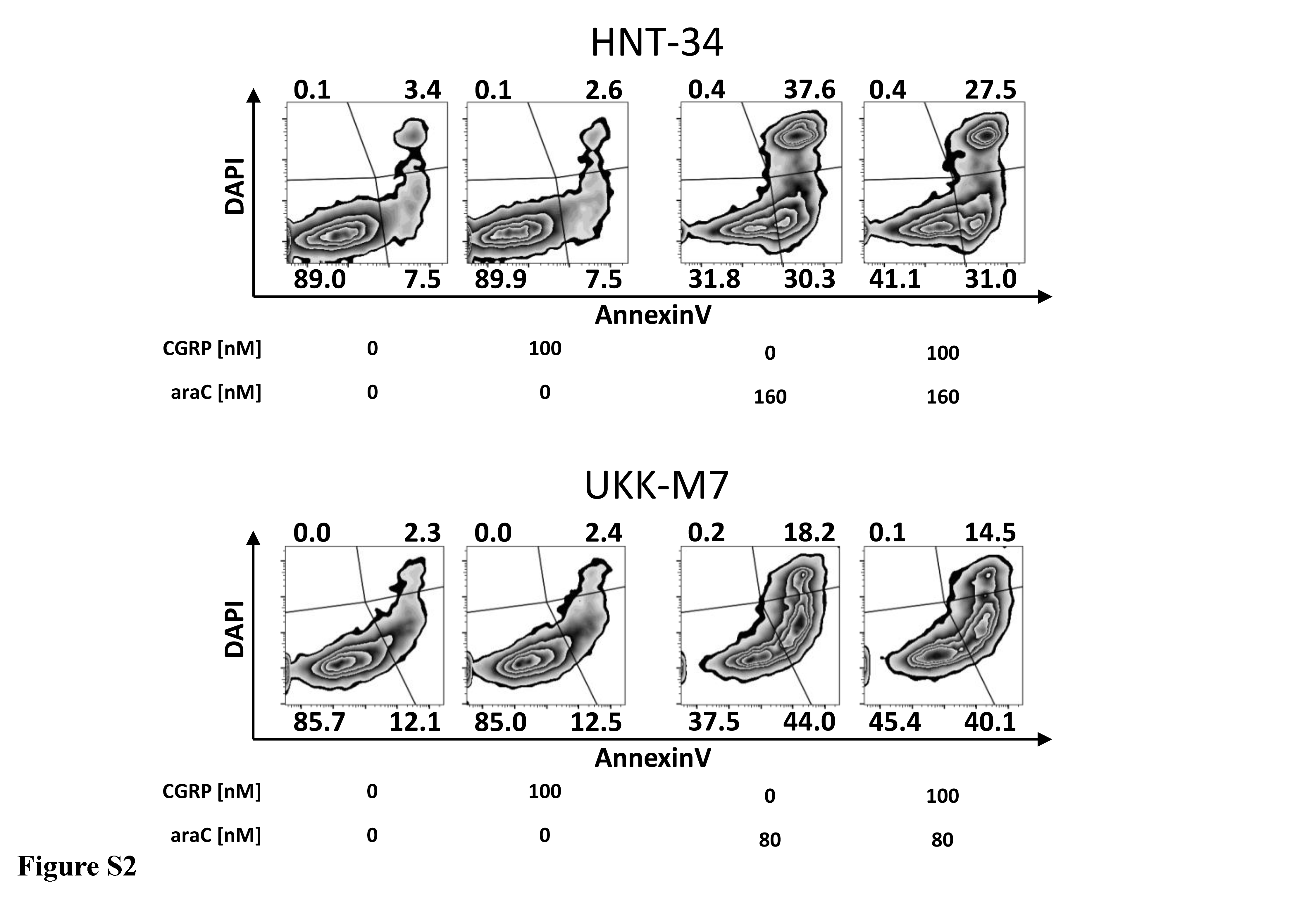

Supplement: Supplementary file 1 [file ijms-20-05826-s001.zip › ijms-616043-supplementary/FigS2.tif]

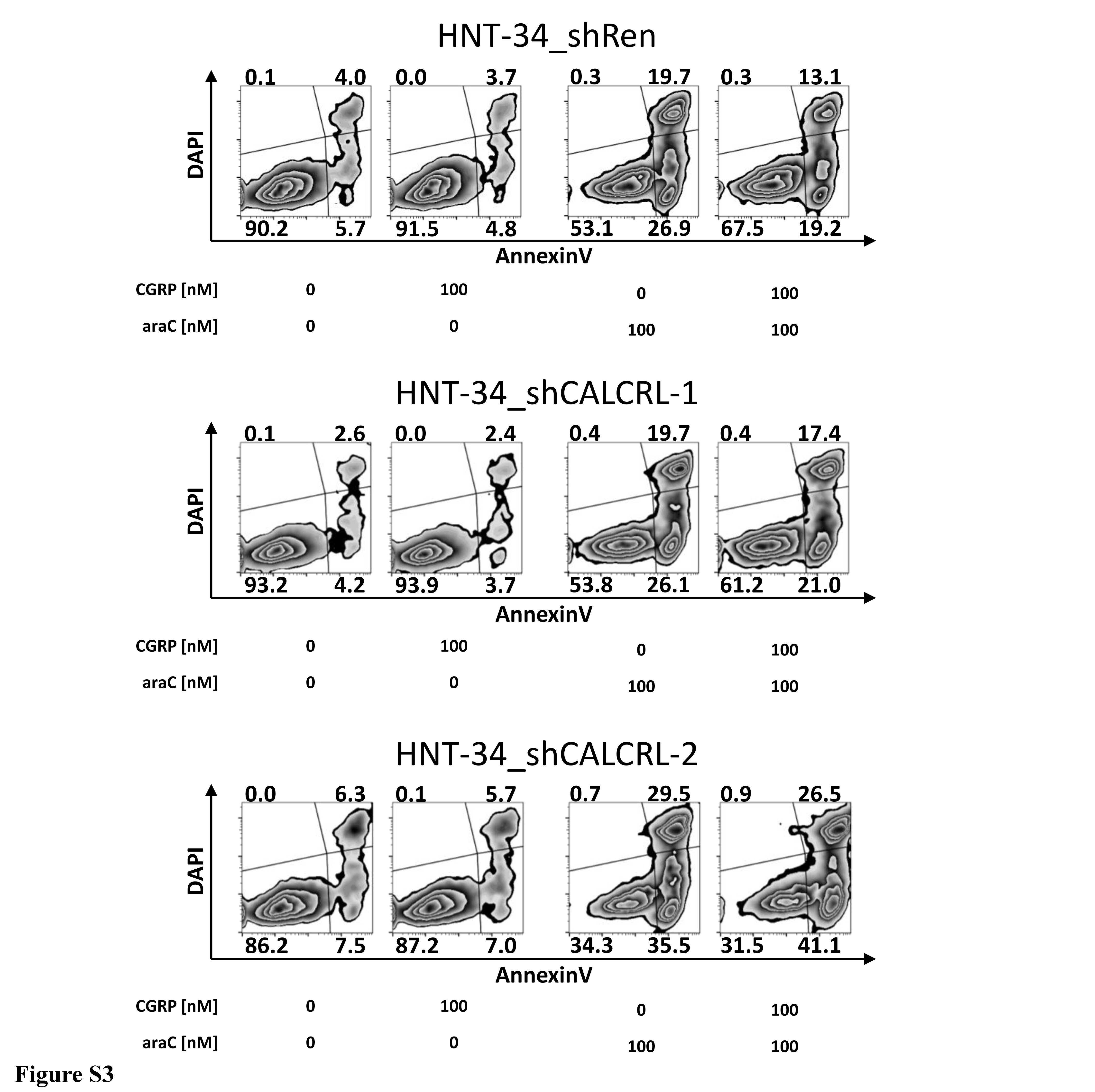

Supplement: Supplementary file 1 [file ijms-20-05826-s001.zip › ijms-616043-supplementary/FigS3.tif]

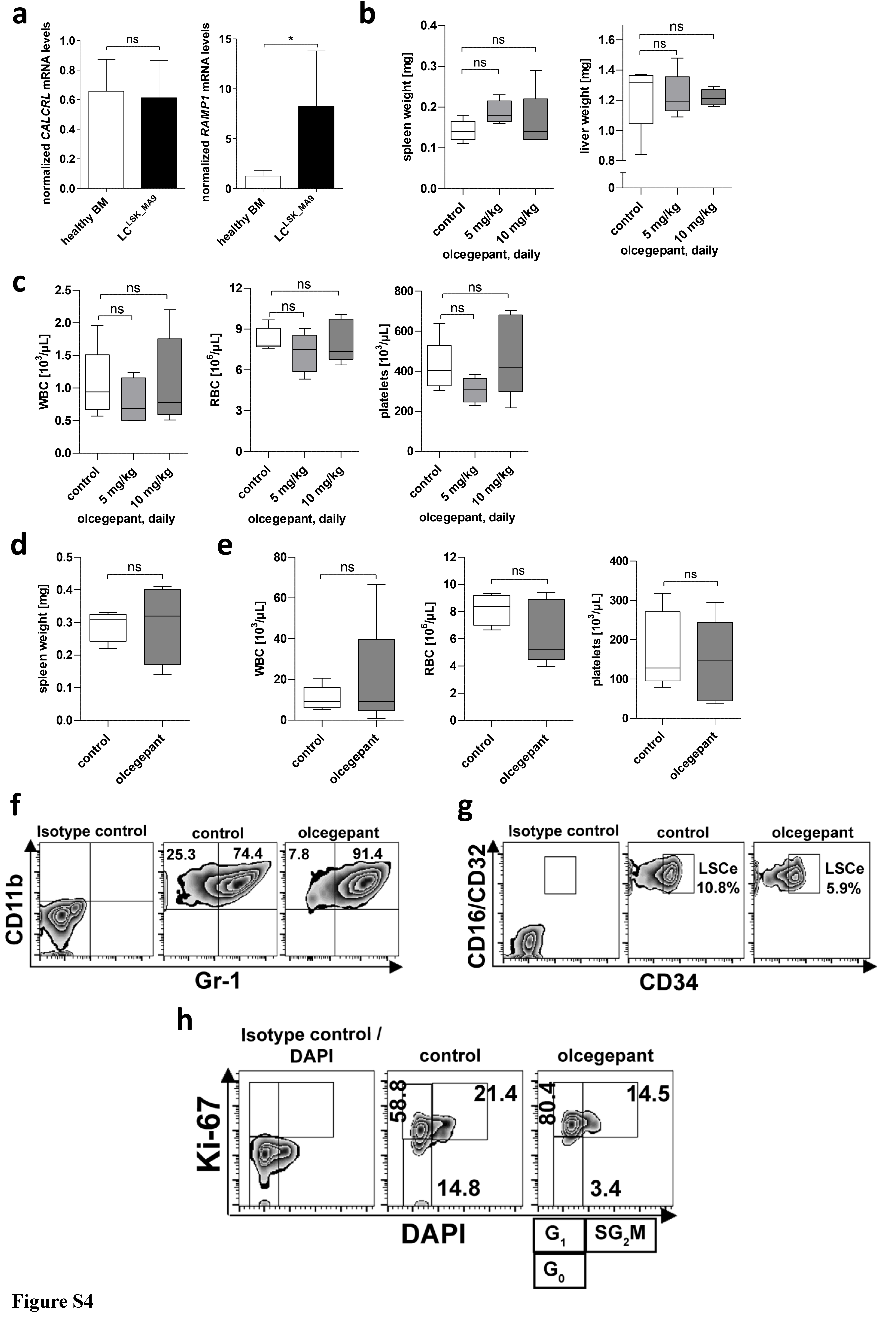

Supplement: Supplementary file 1 [file ijms-20-05826-s001.zip › ijms-616043-supplementary/FigS4.tif]

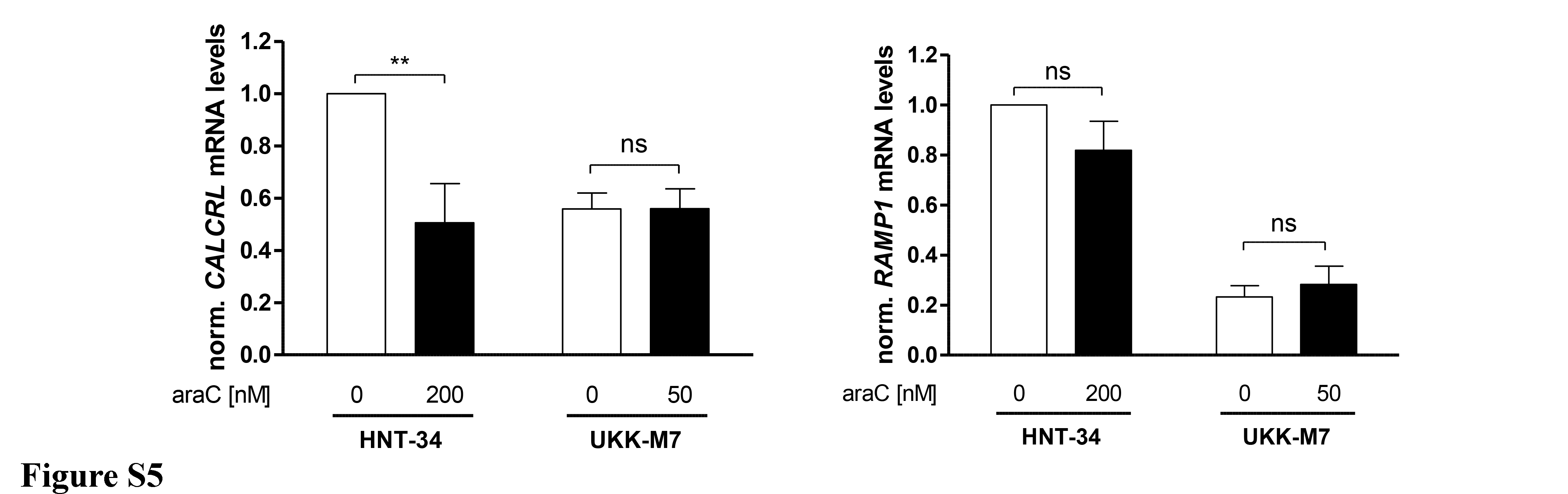

Supplement: Supplementary file 1 [file ijms-20-05826-s001.zip › ijms-616043-supplementary/FigS5.tif]

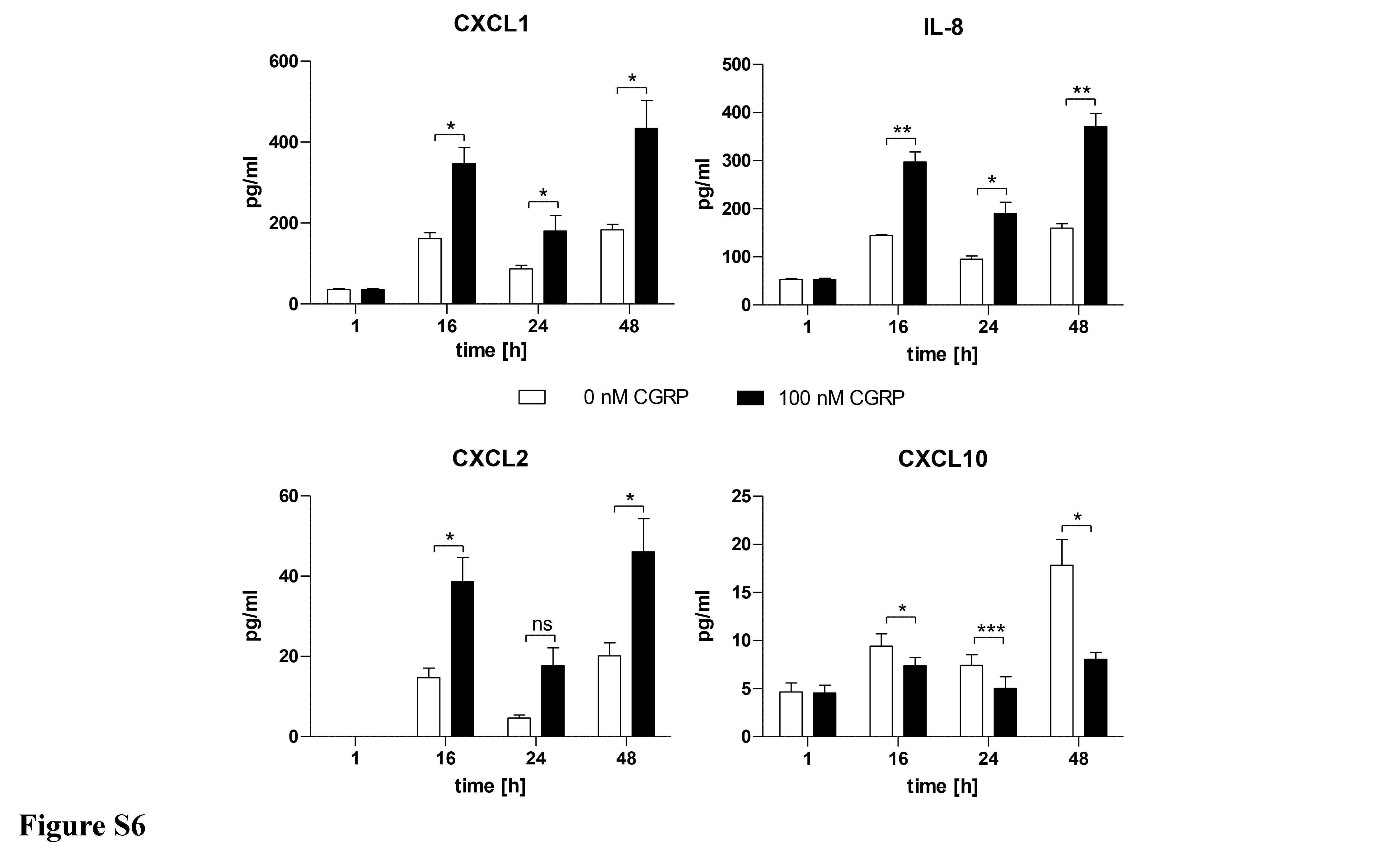

Supplement: Supplementary file 1 [file ijms-20-05826-s001.zip › ijms-616043-supplementary/FigS6.tif]

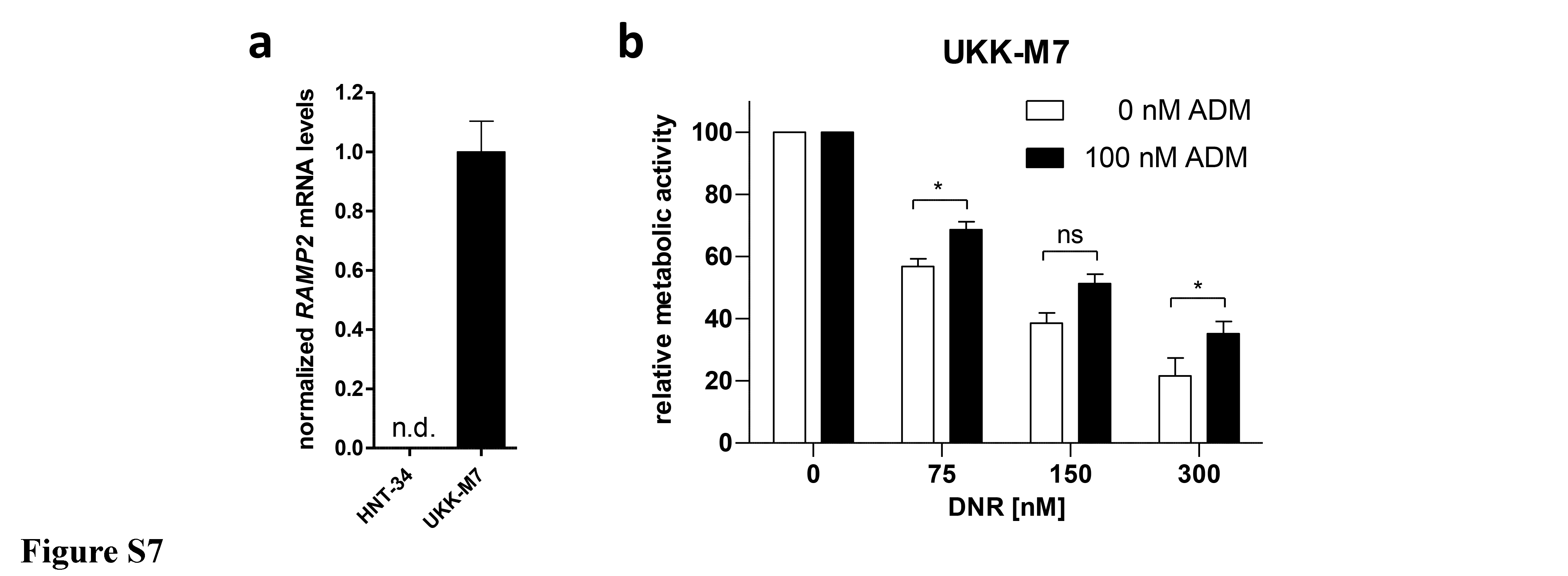

Supplement: Supplementary file 1 [file ijms-20-05826-s001.zip › ijms-616043-supplementary/FigS7.tif]
